# Data2MV - A user behaviour dataset for multi-view scenarios

**DOI:** 10.1016/j.dib.2023.109702

**Published:** 2023-10-20

**Authors:** Tiago Soares da Costa, Maria Teresa Andrade, Paula Viana, Nuno Castro Silva

**Affiliations:** a Centre for Telecommunications and Multimedia, INESC TEC, Porto, Portugal; b Faculty of Engineering, University of Porto, Porto, Portugal; c School of Engineering, ISEP, Polytechnic of Porto, Porto, Portugal

**Keywords:** Multimedia, Multi-view, Head-tracking, Adaptive streaming, View prediction, Deep learning

## Abstract

The Data2MV dataset contains gaze fixation data obtained through experimental procedures from a total of 45 participants using an Intel RealSense F200 camera module and seven different video playlists. Each of the playlists had an approximate duration of 20 minutes and was viewed at least 17 times, with raw tracking data being recorded with a 0.05 second interval. The Data2MV dataset encompasses a total of 1.000.845 gaze fixations, gathered across a total of 128 experiments. It is also composed of 68.393 image frames, extracted from each of the 6 videos selected for these experiments, and an equal quantity of saliency maps, generated from aggregate fixation data. Software tools to obtain saliency maps and generate complementary plots are also provided as an open-source software package. The Data2MV dataset was publicly released to the research community on Mendeley Data and constitutes an important contribution to reduce the current scarcity of such data, particularly in immersive, multi-view streaming scenarios.

Specifications Table

**Table:** 

Subject	Multimedia, Human-Computer Interaction, Software Engineering
Specific subject area	Gaze fixation data for analysis of users’ focus of attention using multi-view video content
Type of data	Table Image (.jpg) Figure
How the data were acquired	Data was acquired from an RGB-D camera module (Intel RealSense F200, installed using a monitor stand) and custom client software (developed in C# with Intel RealSense SDK and tasked with the conversion of gaze fixation data from 3D space to 2D coordinate space). Volunteers were seated in front of a single 24” *Full HD* (1920 *×* 1080) monitor at a distance between 0.8-1 meters and a *FoV* of 56º. Data acquisition was conducted using a color resolution of 1920 *×* 1080 pixels and 30 frames per second. Observers were asked to watch selected playlists, with only one view/video combination being displayed at any given point in time.
Data format	Raw Analysed Filtered
Description of data collection	Gaze fixation data was collected from a total of 45 participants when viewing a total of 7 video playlists composed of 360-degree, multi-view content. Video content was split into six individual perspectives/views, according to cubemap projection. The duration of each playlist was approximately 20 minutes, and participants could visualize each playlist only once in or- der to avoid duplicate data. Outlier data, derived from initial calibration and set-up, was also filtered to discard any potential gaze fixation bias.
Data source location	Institution: Centre for Telecommunications and Multimedia, INESC TEC City/Town/Region: Porto Country: Portugal
Data accessibility	Repository Name: Mendeley Data Data identification number: 10.17632/6ng3ypkyzr.2 Direct URL to data: https://data.mendeley.com/datasets/6ng3ypkyzr/2
Related research article	T. S. Costa, M. T. Andrade, P. Viana, N. C. Silva, *A Dataset for User Visual Behaviour with Multi-View Video Content*, Proceedings of the 14th ACM Multimedia Systems Conference (MMSys ’23), June 7–10, 2023, Vancouver, BC, Canada, 1-7. https://dl.acm.org/doi/10.1145/3587819.3592556

## 1 Value of the Data

• The dataset is composed of 1.000.845 gaze fixations, gathered across 128 experiments, 7 video playlists, and 119 views. Raw and post-processed tracking data are provided as TXT and CSV files, respectively.
• 68.393 image frames (and corresponding saliency maps) are provided as JPG files.
• Data was gathered from a total of 45 participants for the same multi-view video content at different time instances, encompassing all possible gaze fixation variations during preestablished viewing periods. Outlier data (from initial set-up and calibration) was filtered to discard potential fixation bias.
• The dataset contributes to the training, testing, and evaluation of visual attention models, where gaze fixation data is required for the prediction of user gaze behavior and adjustment of content (and/or view) selection, preparation, and distribution.
• Data can be used to evaluate the degree of accuracy of interactive multi-view streaming systems (e.g., selection of new viewpoints based on user viewing behavior data), their effect on system performance (e.g., view switching latency, segment download/buffering), and their overall impact on existing mechanisms (e.g., predictive view selection, multi-view content presentation).
• Due to the scarce availability of datasets targeted for multi-view scenarios, the dataset increases the limited pool of available options with a significant sample of high-resolution image frames, saliency maps, and tracking data (e.g., three-dimensional point clouds) and provides software tools for data management and visualization.

## 2 Objective

The most common option to obtain large sets of gaze fixation data is to use Head-Mounted Devices (HMDs) [1,2]. The availability of such datasets is of high importance to the research community investigating novel solutions for immersive video applications (e.g., 360-degree video streaming [3]). However, using HMDs is not trivial and is often not within reach of everyone. We have thus devised an alternative setup for collecting such data, eliminating the need to utilize HMDs while providing the ability to create datasets useful for a wider range of applications, namely multi-view [4]. Data was collected using an RGB-D camera module (Intel RealSense F200 [5]), in order to capture the attention of the user on the screen when watching multi-view content. In addition to being a non-intrusive, cost-effective solution, the selected RGB-D camera module can be used for interactive tasks without resorting to additional body-centric equipment.

## 3 Data Description

The newly developed dataset described throughout this article encompasses several categories of data, with their own specificities (e.g., video frames, saliency maps, log files). The following section will describe which data can be found in the dataset and, in particular, how such data is organized for better access, comprehension and handling.

### 3.1 Data overview

The dataset is composed of 68.393 image frames, extracted from 360-degree multi-view video content (Fig. 4), and saliency maps, generated from cumulative gaze tracking data collected with the RGB-D camera module (Fig. 5). A representative example of the process of acquiring gaze fixation data during content playback is shown in Figs. 2 and 3. Gaze tracking data was collected with a 0.05 second interval from a total of 45 individuals while viewing 7 video playlists composed by multiple perspectives from original 360-degree content (split into 6 individual views according to cubemap projection). The most significant characteristics of each of the selected videos are presented in Table 1. The gender distribution between participants was almost equal (24 males and 21 females), with every participant being of the same race (white). Ages ranged between 22 and 88 years old, with an average age of 33.33 years and a mode of 25.00 years. 28 of the 45 users (57.7% of the total number of participants) were between 22 and 26 years old. Tracking data was stored using two different file formats: TXT files (raw data, acquired directly through the custom client software) and CSV files (generated from raw data, to enable faster data processing). Raw data files encompass the following data:

**Table 1:** Main properties of the omnidirectional video dataset.

Title	Categories	Video Duration	Avaiable/Selected Views	Capturing Device	Publish Date	Maximum Resolution
Blue Angels 360º Flight	Outdoor, Urban	08:12	6/5	Custom Setup	11/11/2015	3840 *×* 2160
Mercedes-Benz E Class	Outdoor, Urban, Rural, People	03:17	6/4	Custom Setup	20/05/2016	3840 *×* 2160
Rafting on Zambezi River	Outdoor, People, Rural	01:49	6/5	GoPro Fusion 360	07/09/2016	3840 *×* 2160
Carnival of Venice	Outdoor, Urban, People	05:52	6/5	GoPro Fusion 360	08/11/2016	3840 *×* 2160
Amusement Ride	Outdoor, Urban, People	06:15	6/5	GoPro Omni	09/06/2018	3840 *×* 2160
Ancient City of Petra	Outdoor, People, Rural	03:59	6/5	GoPro Omni	20/04/2018	3840 *×* 2160

(1) Anonymized identifier; 2) Age; 3) Gender; 4) Video filename; 5) Video timestamp; 6) Position within the *Hot&Cold* matrix; 7) Raw gaze tracking data from the RGB-D camera, stored under the form of Three Degrees of Freedom (3DoF) [19] orientation data: *pitch, yaw* and *roll*; 8) Three-dimensional positioning data (*x, y*, and *z* axes values), calculated from Euler angles [20] provided by the RGB-D camera for each gaze fixation; 9) Three-dimensional point clouds related to gaze fixations from each participant, as acquired by the RGB-D camera. Additional material is also included with the dataset: an overview of the participant data and video playlist distribution, and complementary software tools developed for data extraction and plot generation. The dataset is 6.73GB in total size and was publicly distributed through Mendeley Data [21,22].

### 3.2 Data organization

**Fig. 1:**
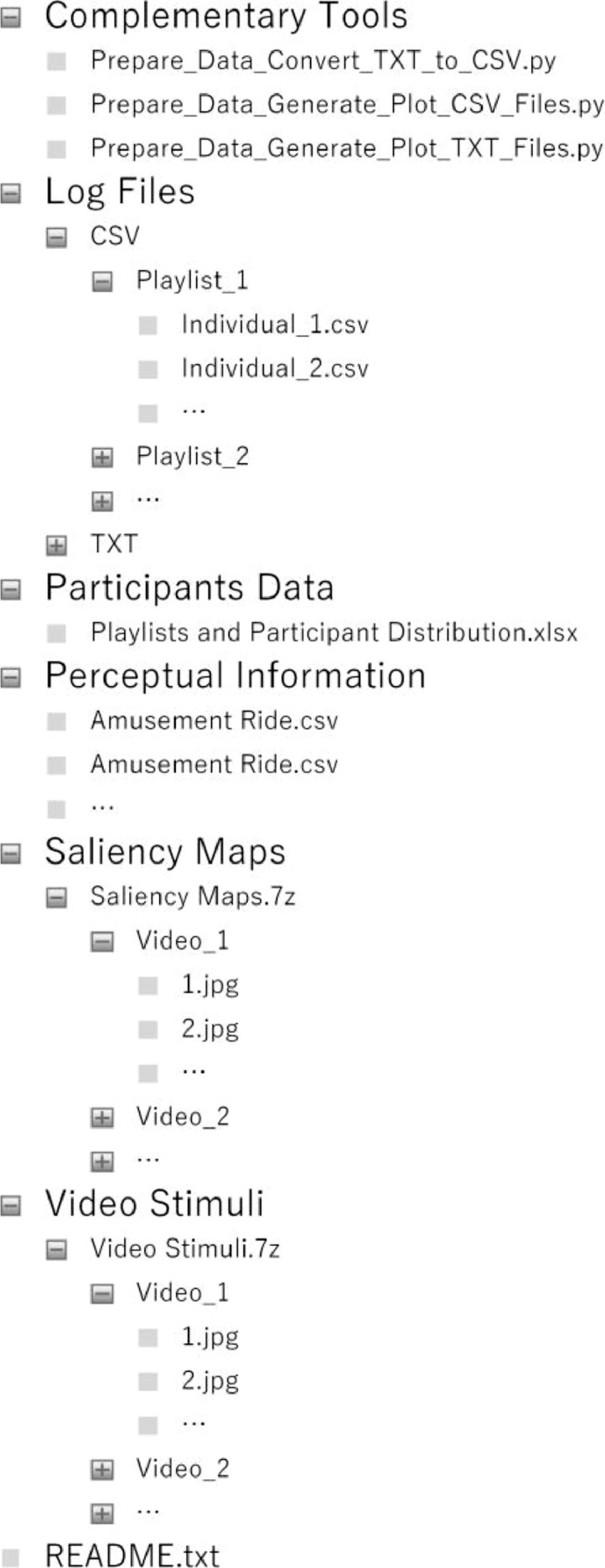
A directory tree depicting the structure of the dataset and its content.

To facilitate access for potential users, data was distributed across six separate directories, as depicted in the directory tree presented in Fig. 1. Contents from each of these directories will now be discussed in detail to provide a clear picture of what type of data can be expected. With regard to the *Video Stimuli* directory, it is composed of a compressed file (*Video Stimuli.7z*) which aggregates image frames extracted from each of the selected 360-degree videos. Views from these videos were spread across individual subdirectories (119 in total), each with a unique identifier (e.g., *Video 1*). Additionally, for each image frame made available, a matching saliency map (a graphical representation of user visual behavior data, as represented in Fig. 5) was also generated from cumulative tracking data. These maps can be accessed in the corresponding directory through the available compressed file (*Saliency Maps.7z*). Participant data is available in the homonyms direc- tory, encompassing an *Excel* spreadsheet (*Playlists and Participant Distribution.xlsx*) where users can consult relevant information from participants (e.g., age, gender), along with participant distribution across available video playlists and views. As for tracking data collected from participants during the experimental procedures, it can be consulted in the *Log Files* directory. Two types of data formats are made available with this dataset: raw data (defined as TXT) and post-processed data (identified as CSV). For this purpose, individual data files are provided for each playlist, view, and participant combination. Spatial and temporal perceptual information, calculated using *siti-tools* [23] for the six 360-degree videos, is provided under the respective directory to further characterize the complexity of the selected video content [24]. Complementary software tools, developed with the sole purpose of facilitating the creation process from this dataset, are also provided for two specific tasks: data preparation (e.g., conversion of raw data to post-processed format) and data visualization (e.g., generation of plots from available data).

### 3.3 Relevance of data

The goal of the dataset is to provide gaze data collected while visualizing multi-view content. The majority of datasets that are currently available for usage (the selection of the most relevant options is presented in Table 2) provide gaze data captured from HMDs while visualizing 360-degree content. Its applicability to multi-view solutions is considered limited due to the specific requirements raised by these types of scenarios (e.g., single- screen visualization, selection of different viewpoints for content presentation). Comparatively, the Data2MV dataset provides gaze data from a wide range of participants (45 users), captured by an RGB-D camera module while visualizing multi-view content. This content was spread across 7 video playlists, with each playlist being viewed a minimum of 17 times. Due to the scarcity of high-resolution multi-view datasets with sufficient content length to provide adequate viewing behavior data, this dataset provides valuable gaze data, which enables its use in interactive multi-view scenarios where elements such as viewing behavior and viewpoint selection are deemed essential.

**Table 2:** Selection of the most relevant datasets for immersive scenarios and applications.

Reference	Title	Publication	Contribution	Comparison
Research				
Jiang et al. [6]	SALICON: Saliency in Context	IEEE CPVR, 2015	360-degree dataset, captured from selected users using a mouse-based paradigm	Simulated natural viewing, reservations should be made with regards to multi-view scenarios
Corbillon et al. [7]	360-Degree Video Head Movement Dataset	ACM MMSys, 2017	Head movement dataset, captured from users watching 360-degree videos	Reduced set of video content, HMD-based acquisition, limited application for multi-view scenarios
Rai et al. [8]	A Dataset of Head and Eye Movements for 360 Degree Images	ACM MMSys, 2017	Dataset with sixty 360-degree images, tools, and guidelines for evaluation	Image-based gaze data acquisition, unsuitable duration for longer experiments (25 seconds)
Chenglei et al. [9]	A Dataset for Exploring User Behaviors in VR Spherical Video Streaming	ACM MMSys, 2017	User behavior dataset, focused on VR spherical video streaming	360-degree video content, HMD-based acquisition, requires adaptation for multi-view applications
Fan et al. [10]	Fixation Prediction for 360° Video Streaming in Head-Mounted Virtual Reality	ACM NOSSDAV, 2017	360-degree dataset for HMD-based VR streaming scenarios	Limited set of participants (25) and videos (10), gaze data collected strictly using HMDs
Lo et al. [11]	Content and Sensory Datasets from 360-degree Video Streaming Viewers with HMDs	ACM MMSys, 2017	HMD-based 360-degree dataset for VR applications	Limited set of videos (10), HMD-based acquisition, requires adaptation for multi-view scenarios
Maugey et al. [12]	FTV360: a Multiview 360-degree Video Dataset with Calibration Parameters	ACM MMSys, 2019	Free viewpoint dataset, captured using arrays of omnidirectional cameras	Synchronized scene-based video acquisition, does not contain any gaze tracking data
Nasrabadi et al. [13]	A taxonomy and dataset for 360° videos	ACM MMSys, 2019	User viewing behavior data while visualizing 360-degree videos	Large-scale dataset, provided viewport traces require adaptation for multi-view applications
Miller et al. [14]	Personal identifiability of user tracking data during observation of 360-degree VR video	Scientific Reports, 2020	User identifiability dataset under VR viewing circumstances	Significant user pool (511), unsuitable video duration for longer experiments (20 seconds)
Guo et al. [15]	A new free viewpoint video dataset and DIBR benchmark	ACM MMSys, 2022	RGB-D data captured from multiple angles in free viewpoint scenarios	Depth-based multi-view dataset, purposely developed for volumetric video streaming
Jin et al. [16]	Where Are You Looking?: A Large-Scale Dataset of Head and Gaze Behavior for 360º Videos	ACM MM, 2022	Dataset containing users’ head and gaze behaviors in 360-degree scenarios	HMD-based, reduced content (3 playlists/9 videos), limited application for multi-view solutions

Industry				

CIVIT [17]	360-degree Stereo Video Test Datasets	CIVIT, 2019	360-degree stereo video dataset for immersive applications	VR-based dataset, requires adaptation for multi-view, does not contain any gaze tracking data
Miraikan [18]	Miraikan 360-degree Video Dataset	Miraikan, 2023	Dataset composed by 4K/60fps 360-degree video, captured inside Miraikan	Large-scale scene-based 360-degree dataset, does not contain any gaze tracking data

## 4 Experimental Design, Materials and Methods

Experimental procedures conducted during the creation of the dataset required a specific set of conditions in order to conclude such a task with success. For example, the selection of appropriate hardware and multi-view video content to enable meaningful data acquisition or the development of a custom software solution for accu- rate data processing and analysis. The following section will delve in detail into each of these components.

### 4.1 Hardware setup

**Fig. 2:**
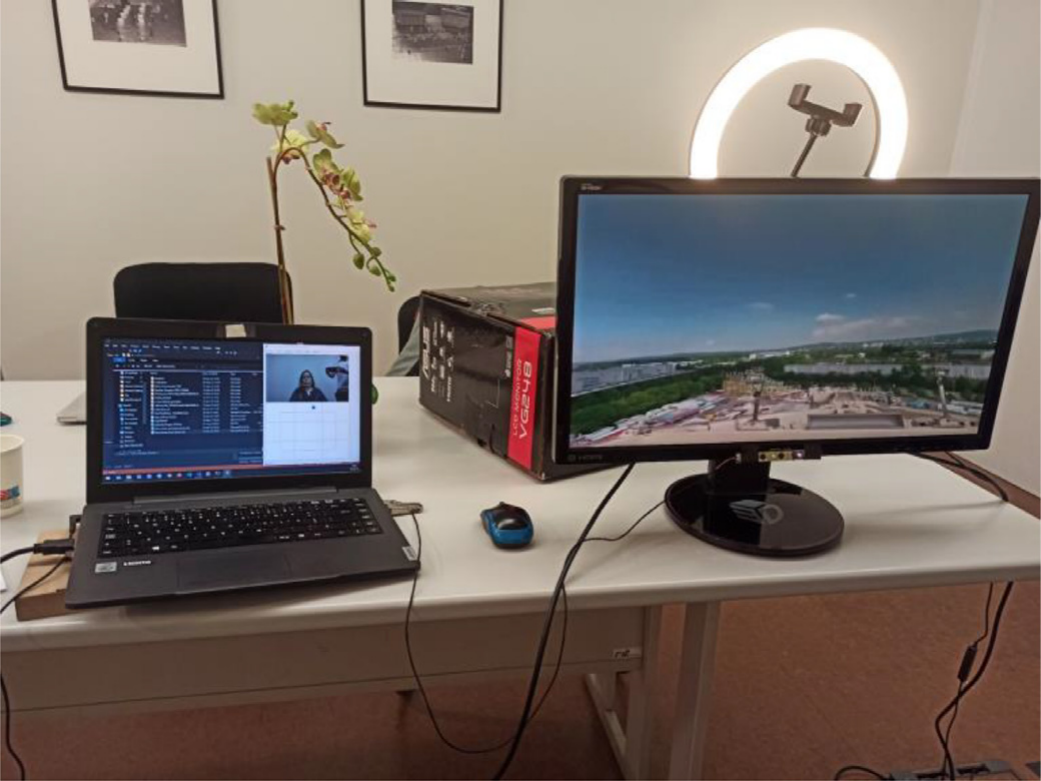
The RGB-D camera setup, used for collection of gaze data from users.

An experimental setup, composed of a laptop computer, a computer monitor, and an RGB-D camera module (as visible in Fig. 2), was defined for the purpose of acquiring gaze fixation data. With regard to the RGB-D camera module, an Intel RealSense F200 camera was physically installed below the computer monitor (using a monitor stand) and connected to an HP Elitebook 940 G2 laptop computer using an USB-3.0 to USB-3.0 connection. The RGD-D camera module is composed of five core elements: a color sensor, an infrared sensor (IR), an IR laser projector, an image processor, and a stereo microphone [25]. For the development of this dataset, solely the color sensor, IR sensor, and IR laser projector were used to capture depth data in Full HD resolution (1920 x 1080) at 30 frames per second. Depth data was acquired from users using a three-step process [5]: 1) An IR laser projector emits a structured light pattern; 2) An IR sensor detects reflected light patterns on objects or individuals; 3) 3D surfaces are reconstructed from reflected patterns and stored as point clouds. According to the specifications from Intel, users must be situated within the recommended range of 0.2–1.2 meters in order to achieve optimum depth accuracy values (prior literature [5] confirms that an average depth accuracy between 1.46 and 1.53mm RMS is achieved by the Intel Realsense F200 under similar conditions). A 24” Full HD (1920 × 1080) computer monitor was installed for the presentation of multi-view content to selected observers at a distance situated between 0.8 and 1.0 meters and with a *FoV* of approximately 56º.

### 4.2 Client software

**Fig. 3:**
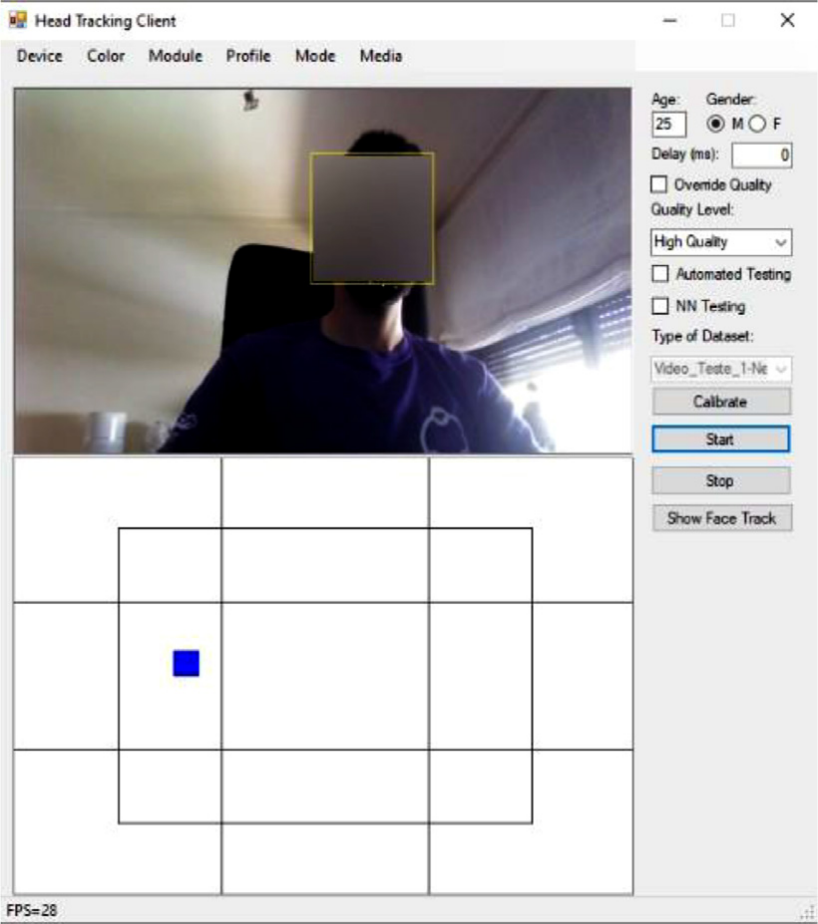
Client software, tracking a user viewing multi-view content (face blurred for anonymization purposes).

To collect gaze data in real-time, a custom client software solution was developed in C#, combining Intel RealSense SDK tracking features [26] with Windows Media Player video playback capabilities. An overview of the workflow used for the collection of gaze tracking data using the client software can be visualized in Fig. 6. The workflow is initiated with the obligatory calibration procedure, conducted prior to any viewing session: users were asked to follow a red dot presented across the screen in order to confirm that tracking data accu- rately represented their facial movements. After this initial procedure was concluded with success, multi-view content was presented to users according to the video composition included within each playlist. Selected multi- view content was downloaded from the dataset storage and presented to users in full screen (using Windows Media Player components for such purposes). During the presentation of multi-view content, gaze tracking data (e.g., three-dimensional point clouds, Euler angles) was simultaneously collected in real-time, synchro- nized with multi-view content data (e.g., timestamps), and continuously stored in the form of raw data in TXT files. Additionally, the conversion of gaze fixation data from a three-dimensional space into a two-dimensional coordinate space was also conducted by the client software solution: *x, y* and *z* axes values were computed from *pitch, yaw* and *roll* values previously acquired and stored as Euler angles [20] by the RGB-D camera module. These computed values were projected into the *Hot&Cold* matrix structure [4] (considering camera parameters such as center calibration, variable *FoV* and distortion [27]), delivering a graphical representation of users’ viewing behavior in real-time (Fig. 3), during content playback. After the process of collecting gaze data was concluded, an additional set of post-processing tasks were also conducted, as depicted in the workflow presented throughout Fig. 7: 1) For each available multi-view video content used for gaze data acquisition, a predefined set of image frames was extracted using FFMpeg tools based on its overall relevance (e.g., keyframes); 2) For each of the extracted image frames, corresponding saliency maps were generated us- ing the cumulative gaze tracking data previously collected from the participants; 3) Image frames and related saliency maps were stored in the corresponding playlist/video directory, available at the dataset storage location.

**Fig. 4:**
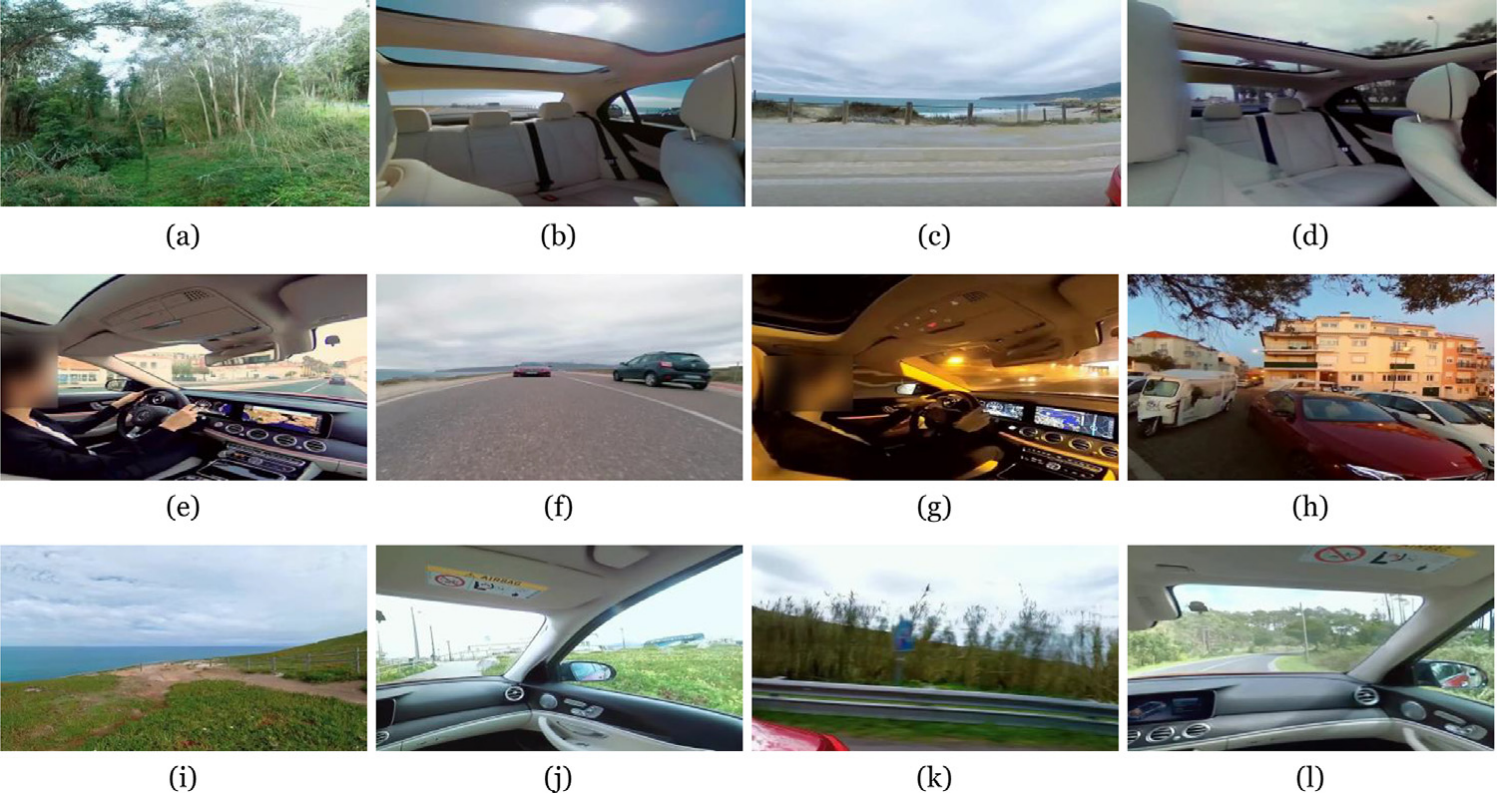
A representative set of video frames from available views on each of the selected playlists.

**Fig. 5:**
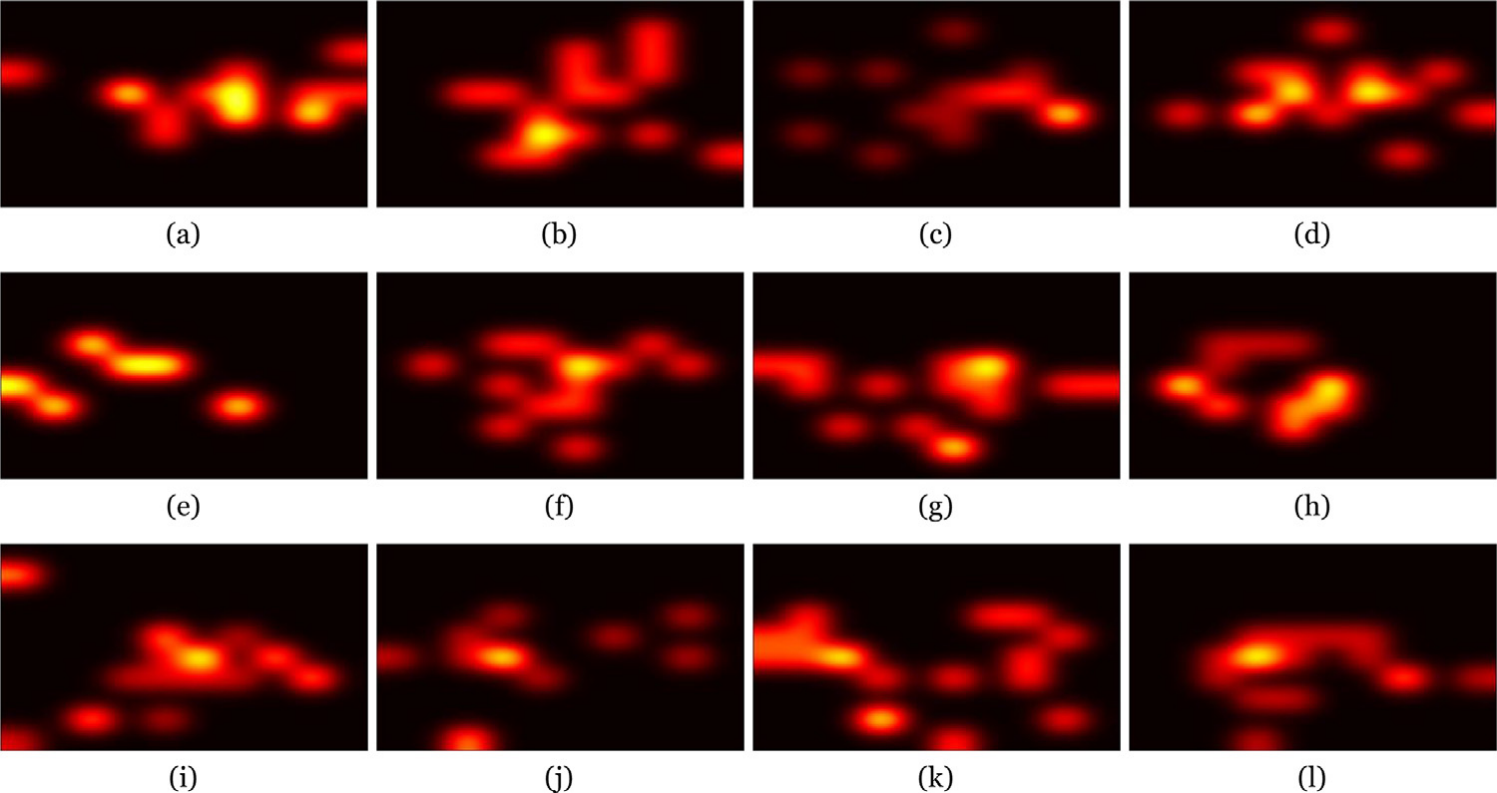
A representative set of saliency maps from corresponding video frames.

**Fig. 6:**
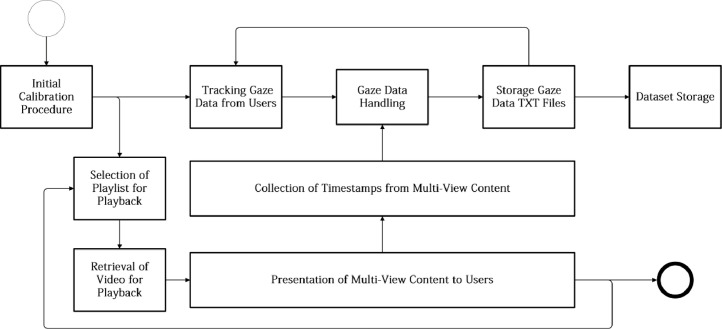
Sequence diagram detailing the process of collecting gaze data from participants.

**Fig. 7:**
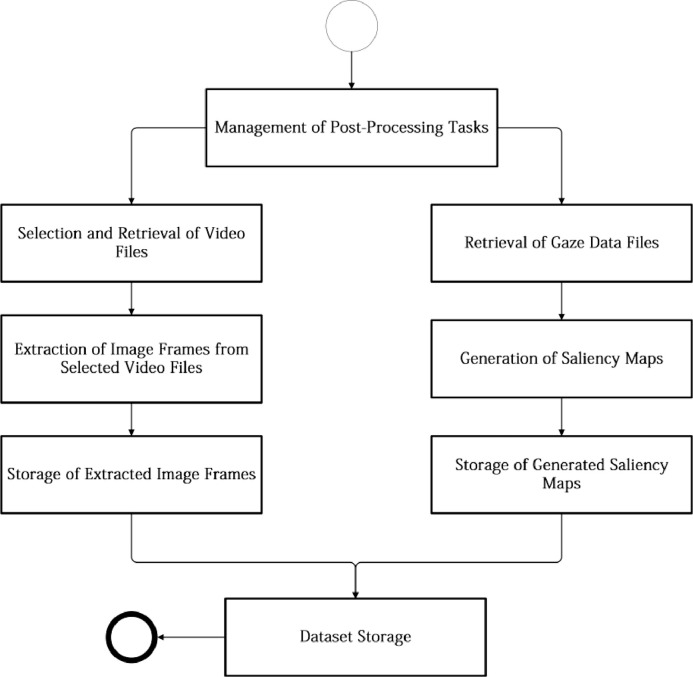
Sequence diagram detailing relevant post-processing tasks applied collected tracking data.

### 4.3 Test stimulus

To acquire gaze fixation data from participants, six 360-degree videos (available under Creative Commons copyright) were selected for playback purposes (video specifications can be consulted in Table 1). These were originally presented in equi-rectangular format and encoded in 4K resolution (3840 2160 pixels), with frame-rates ranging between 24 and 30 frames per second. To present each perspective according to cubemap projection, videos were split into six individual views (each with a resolution of 1280 1080 pixels). From the 36 views created, 7 were discarded due to a lack of visual interest in its content. Smaller portions of the remaining views (13 in total) were also discarded to allow for a better distribution of views among selected playlists. To characterize content from selected videos, four categories were considered: Outdoor (e.g., natural landscapes), Urban (e.g., urban objects and architecture), Rural (e.g., non-urban environments), and People (e.g., human presence). Additionally, Temporal Perceptual Information (TI) and Spatial Perceptual Information (SI) data were also computed for each of the videos by resorting to *Sobel* filters (3 *×* 3 pixels) [28].

## Ethics Statements

Being a free-for-all experimental activity, participants demonstrated their interest at their own discretion. To guarantee that General Data Protection Regulation (GDPR) guidelines [29] were strictly followed, the following procedures were conducted: 1) Request permission from participants to collect, store, analyze, and publish collected data; 2) Restrict data gathered from participants during the experimental procedure; 3) Subject data to anonymization techniques (e.g., removal of personal identification data).

## CRediT authorship contribution statement

**Tiago Soares da Costa:** Conceptualization, Methodology, Software, Data curation, Visualization, Writing – review & editing. **Maria Teresa Andrade:** Supervision, Writing – review & editing. **Paula Viana:** Supervision, Writing – review & editing. **Nuno Castro Silva:** Software, Investigation, Writing – original draft.

## Data Availability

Data2MV - A User Behaviour Dataset for Multi-View Scenarios (Original data) (Mendeley Data).
